# Research on a Novel AC/DC Hybrid Microgrid Based on Silicon Controlled Converters and Polarity Reversal Switches

**DOI:** 10.3390/s25061766

**Published:** 2025-03-12

**Authors:** Yang Lei, Fan Yang, Jiaxuan Ren, Zhichun Yang, Xinchen Wang, Qianchen Chen, Xuan Jin, Shaorong Wang

**Affiliations:** 1 Electric Power Research Institute of State Grid Hubei Electric Power Co., Ltd., Wuhan 430077, China; leiyang1019@163.com (Y.L.); yangf_82@163.com (F.Y.); yangzhichun3600@163.com (Z.Y.); 2 School of Electrical and Electronic Engineering, Huazhong University of Science and Technology, Wuhan 430074, China; m202271896@hust.edu.cn (X.W.); m202271885@hust.edu.cn (X.J.); wsrwy96@vip.sina.com (S.W.); 3 Haomai Electric Power Automation Co., Ltd., Wuhan 430074, China; 13607131283@163.com

**Keywords:** silicon controlled converter, polarity reversal switch, AC/DC hybrid microgrid, distributed energy

## Abstract

In order to reduce the economic costs, enhance the efficiency, and improve the structural stability of microgrids, this paper proposes a novel AC/DC hybrid microgrid structure. This structure, based on Silicon Controlled Converters (SCCs) and Polarity Reversal Switches (PRSs), enables bidirectional power flow and provides a low-cost and straightforward control solution. This paper elaborates on the overall control strategy of the microgrid under different states of the PRS and introduces the control logic of the Current Reversible Chopper (CRC) circuit. For typical daily scenarios across the four seasons, where wind and photovoltaic (PV) power generation outputs and load demands vary, this study combines sampled data to investigate the coordinated configuration scheme of wind energy, PV energy, and energy storage within the microgrid, and analyzes the state changes in the PRS. Furthermore, this paper conducts simulation analysis of the microgrid under different states of the PRS and during the switching process of the PRS, verifying the feasibility of the proposed new structure. Finally, this paper compares the proposed structure with traditional microgrid structures in terms of economics, system efficiency, and structural stability, and analyzes the impact of this structure on the frequency, inertia, and multi-energy interaction of the system.

## 1. Introduction

With a heightened awareness of environmental protection, advancements in renewable energy technologies, and the pressing necessity for governments to tackle climate change, the installed capacity of renewable energy continues to climb steadily [1]. However, the inherent uncertainty and intermittency of renewable energy sources pose significant challenges [2], necessitating energy storage systems (ESSs) to guarantee the stable operation of power systems during their integration and consumption processes [3,4]. Microgrids have emerged as a promising solution to facilitate the seamless integration of renewable energy and energy storage into the power system [5,6]. Microgrids can operate in two main modes: island mode and grid-connected mode [7]. In island mode, microgrids operate independently of the external grid, while in grid-connected mode, microgrids interact with the external grid to perform functions such as peak shaving and valley filling [8,9].

Microgrids can be further categorized based on their voltage type: AC microgrids, DC microgrids, and AC/DC hybrid microgrids. AC microgrids, being the most common and traditional form, leverage the prevalence of AC in existing power sources and electrical devices, making their implementation relatively straightforward. However, AC microgrids confront challenges related to system synchronization and reactive power regulation [10]. DC microgrids, on the other hand, circumvent these issues [11] and can directly interface with the DC output of renewable energy sources, such as solar and wind [12]. However, DC microgrids cannot be easily connected to the external grid, limiting their flexibility. Therefore, AC/DC hybrid microgrids, which combine the strengths of both AC and DC systems, have emerged as an optimal solution, offering enhanced flexibility and efficiency in energy distribution [13,14].

Voltage Source Converters (VSCs) have gained widespread application and are recognized as a promising solution for integrating large-scale renewable energy sources into the grid [15,16], particularly in offshore wind power [17] and photovoltaic (PV) generation systems [18]. In microgrids, VSCs are frequently used to transfer excess renewable energy from the DC side to the AC side or external grid. For instance, a multifunctional VSC capable of maximizing power extraction and suppressing harmonics is presented in [19], while [20] investigates the efficiency and power density of modular multilevel converters in AC/DC hybrid microgrids. Furthermore, a multi-time-scale adaptive droop control algorithm for VSCs to manage power distribution in AC/DC hybrid microgrids is proposed in [21].

Although VSCs are fully controllable devices capable of bidirectional power transfer, the increasing deployment of AC/DC hybrid microgrids raises concerns regarding cost and the complexity of control strategies. Numerous studies have explored cost optimization schemes for AC/DC microgrids [22,23,24] to enhance their economic performance. However, these approaches often rely on complex control algorithms that can increase system overhead and reduce overall efficiency. To address these issues and simplify control mechanisms, this paper proposes a novel AC/DC hybrid microgrid structure that integrates the Silicon Controlled Converter (SCC) for both rectification and active inversion [25], coupled with a Polarity Reversal Switch (PRS). This innovative design aims to diminish both system complexity and cost while preserving efficient power management.

**Figure 1:**
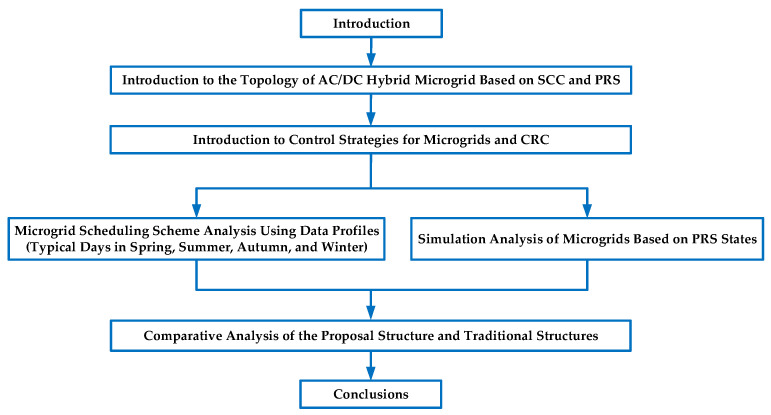
Flowchart to clarify the relationships among each section.

The remainder of this paper is organized as follows: Section 2 introduces the topology and control strategies of the proposed microgrid. Section 3 discusses the coordinated configuration scheme and switch control strategies for typical days. Section 4 presents simulation results under various states of PRS. Section 5 compares the novel microgrid with existing and traditional microgrid structures. Section 6 presents the conclusions and limitations of the research. The relationships between each section can be clarified through the flowchart shown in Figure 1.

The main contributions of this paper are listed as follows:

1. This paper proposes a novel AC/DC hybrid microgrid structure based on the SCC and PRS, with detailed control strategies for different PRS states.
2. This paper studies the wind–PV-ESS coordinated configuration scheme for typical days throughout the four seasons, discusses the control strategy of PRS for these typical days, and verifies the feasibility of the proposed novel microgrid structure through simulations.
3. This paper enhances the energy efficiency of the microgrid by reducing conversion losses, stabilizing voltage, and improving the absorption capacity of renewable energy, ultimately resulting in cost savings and reduced reliance on external grids.

## 2. Principle of the AC/DC Hybrid Microgrid Based on the SCC and PRS

This paper proposes a new AC/DC hybrid microgrid structure based on the SCC and PRS. The proposed structure offers low cost, simple control, and efficient renewable energy integration, while also allowing power to be fed back to the external grid.

In this section, the topology and control strategies of the AC/DC hybrid microgrid based on the SCC and PRS are introduced.

### 2.1. Topology of the AC/DC Hybrid Microgrid Based on the SCC and PRS

**Figure 2:**
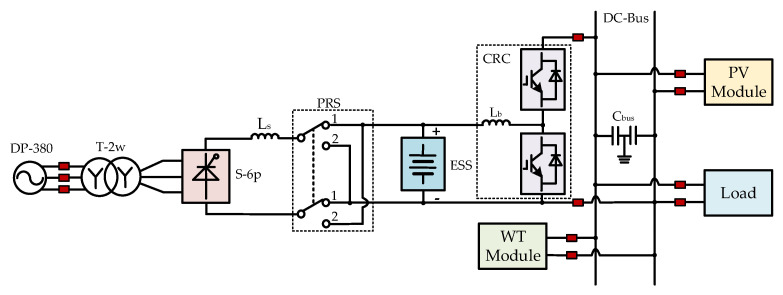
Topology of the AC/DC microgrid based on a six-pulse Silicon Controlled Converter (SCC) and a Polarity Reversal Switch (PRS).

The proposed topology of the AC/DC hybrid microgrid based on the SCC and PRS is shown in Figure 2. The microgrid primarily consists of the following components: the AC power source DP-380, the dual-winding transformer T-2w, the SCC (labeled as S-6p in Figure 2), the PRS, the ESS, the Current Reversible Chopper (CRC) circuit, which includes the lower IGBT T1 and diode D1, and the upper IGBT T2 and diode D2, the PV power module, the wind power module, and the load module.

**Figure 3:**
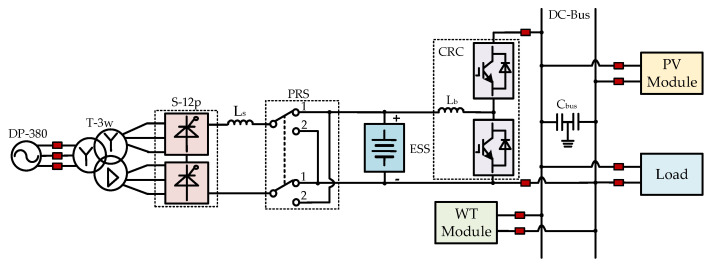
Topology of the AC/DC microgrid based on a twelve-pulse SCC and a PRS.

Considering the power quality issues introduced by the SCC, the six-pulse converter S-6p can be replaced with the twelve-pulse converter S-12p, and the dual-winding transformer T-2w can be replaced with the three-winding transformer T-3w, as shown in Figure 3.

### 2.2. Control Strategy of the AC/DC Hybrid Microgrid Based on the SCC and PRS

In the AC/DC hybrid microgrid proposed in this paper, based on the SCC and PRS, the states of the PRS, which are also the states of the microgrid, are categorized into three types, as shown in Table 1: state 0 occurs when no trigger pulse is applied to the SCC, meaning no power flows through the PRS. State 1 occurs when the SCC is in rectification mode, i.e., when the PRS is in position 1 and the trigger angle α is less than 90°. State 2 occurs when the SCC is in active inversion mode, i.e., when the PRS is in position 2 and the trigger angle α is greater than 90°. The operational states of the microgrid considered in this paper are classified based on the range of the state of charge (SOC) of the ESS. The absence of one of the renewable sources, such as PV energy or wind energy, does not directly result in an abrupt change in the SOC of the ESS. Therefore, it is not treated as an additional state here.

State 0 corresponds with the situation where the SOC of the ESS is within the set reasonable range [SOC_min_, SOC_max_]: if the output of wind and PV generation meets the load demand exactly, then both renewable energy sources supply power, and the ESS do not operate (no trigger pulses are provided to the CRC and the SCC, and the position of the PRS remains unchanged, such as in position 1 or position 2). If the output of wind and PV generation cannot meet the load demand, the Boost circuit formed by T_1_ and D_2_ in the CRC circuit shown in Figure 3 supplements the energy required by the load. If the output of wind and PV generation is excessive, the Buck circuit formed by T_2_ and D_1_ charges the ESS. The power balance equation for this state is shown in (1). In the equation, *P_PV_*, *P_WT_*, *P_ESS_*, and *P_Load_* represent the power output from PV, wind, the energy storage system, and the load power demand, respectively. If the power loss rate of the load, denoted by *β*, is considered, it satisfies (2).

(1) $$P_{PV}+P_{WT}+P_{ESS}=P_{Load}$$

(2) $$P_{PV}+P_{WT}+P_{ESS}=P_{Load}/\left(1-\beta \right)$$

State 1 corresponds to the situation where the SOC of the ESS is less than SOC_min_: the SCC is in rectification mode and is used to supplement the required power of the DC-side load. The power balance equations for this state are shown in (3) and (4). In the equations, *P_EG_* represents the power flowing through the PRS.

(3) $$P_{PV}+P_{WT}+P_{EG}=P_{Load}$$

(4) $$P_{PV}+P_{WT}+P_{EG}=P_{Load}/\left(1-\beta \right)$$

State 2 corresponds to the situation where the SOC of the ESS is greater than SOC_max_: The SCC operates in active inversion mode, delivering power from the microgrid back to the main grid. The power balance equations for this state are shown in (5) and (6).

(5) $$P_{PV}+P_{WT}=P_{Load}+P_{EG}$$

(6) $$P_{PV}+P_{WT}=P_{Load}/\left(1-\beta \right)+P_{EG}$$

**Figure 4:**
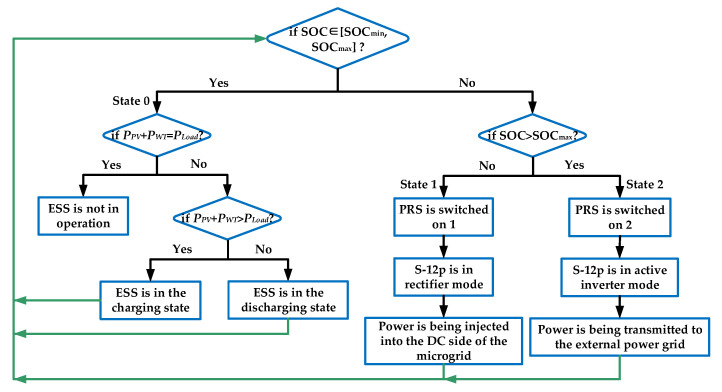
Control strategy of the AC/DC hybrid microgrid based on the SCC and PRS.

Based on the above content, the main control strategy of this microgrid structure can be summarized in the flowchart shown in Figure 4.

**Figure 5:**
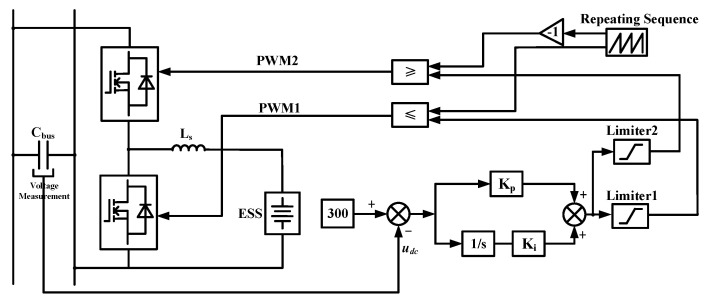
Control logic used in the Current Reversible Chopper (CRC) circuit.

The charging and discharging operations of the microgrid by ESS are primarily controlled by the CRC circuit, with the control logic of this circuit illustrated as shown in Figure 5.

## 3. Microgrid Scheduling Scheme Analysis Using Data Profiles

For the novel AC/DC hybrid microgrid structure proposed in this paper, based on the SCC and PRS, data on PV power generation, wind power generation, and DC-side load consumption were selected for typical days in spring, summer, autumn, and winter (data for one day of each season were selected). The data were sourced from the electricity consumption statistics of a prefecture-level city in Northeast China in 2020. The load profile, which is also based on sampling data, represents the overall consumption of both residential and industrial sectors. The installed capacity of wind turbines is 482 kW, and the installed capacity of PV generators is 57.3 kW. These data were used to study microgrid scheduling and the control strategy of PRS. The energy storage capacity is set to 3 MWh, the initial SOC is 0.7, and the normal SOC range is [0.4, 0.9]. The load loss rate is set to 5%. As discussed in Section 2.2, when the SOC of the ESS is outside the normal range, it is necessary to control the PRS to interact with the external grid for energy exchange.

### 3.1. Typical Day in Spring

**Figure 6:**
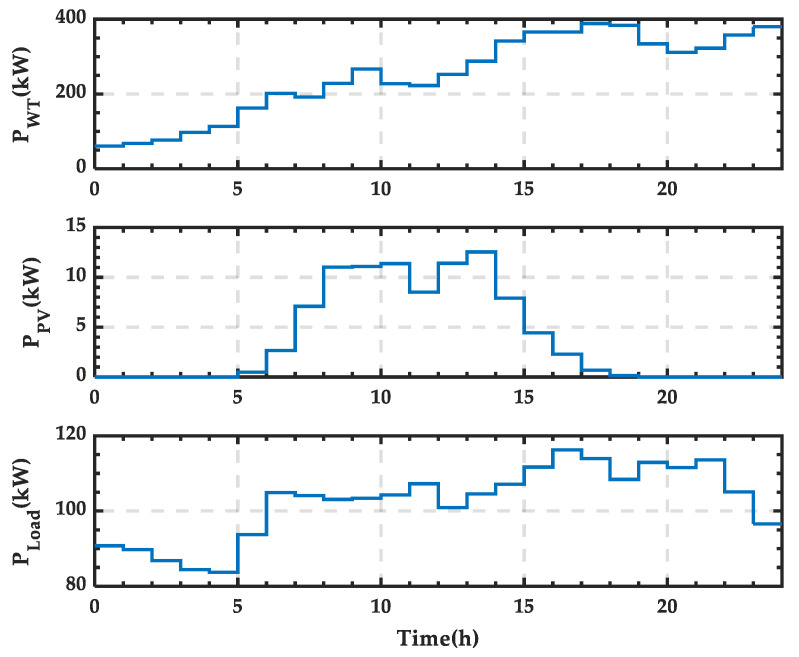
Wind and photovoltaic (PV) power output and load power curves for a typical day in spring.

In this subsection, wind power output *P_WT_*, PV power output *P_PV_*, and load power consumption *P_Load_* data from a typical day in April are selected as the study objects. The day is divided into 24 time periods, and the power distribution across these periods is shown in Figure 6. It can be observed that wind power output generally increases throughout the day, PV power output is higher around midday, and load power consumption is higher between 15:00 and 22:00, reaching a low point during the early morning hours.

**Figure 7:**
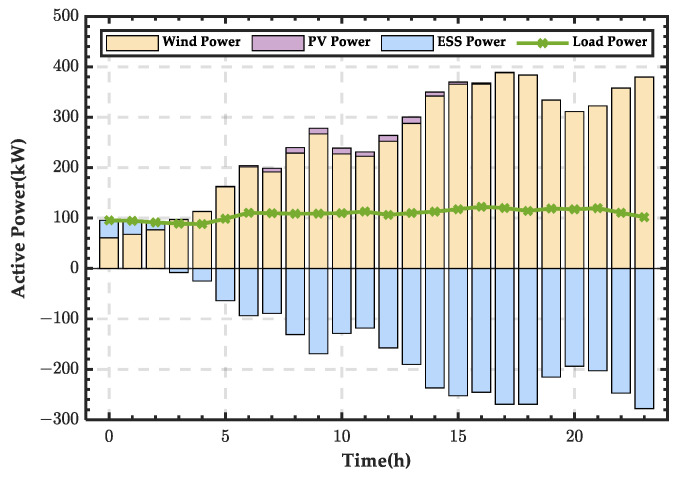
Wind–PV-ESS coordinated configuration scheme for a typical day in spring.

Based on the power balance equations of the microgrid, the wind–PV-ESS coordination scheme for a typical day in spring can be obtained, as shown in Figure 7. In the figure, a positive energy storage value indicates that the ESS is in a discharging state, supplementing the energy deficit in the microgrid. A negative energy storage value indicates that the ESS is in a charging state, storing the excess wind and PV energy in the microgrid. It can be observed that, due to abundant wind resources in spring and higher wind power output, the ESS remains in a charging state for most of the day, only discharging during the time period from 00:00 to 02:00.

**Figure 8:**
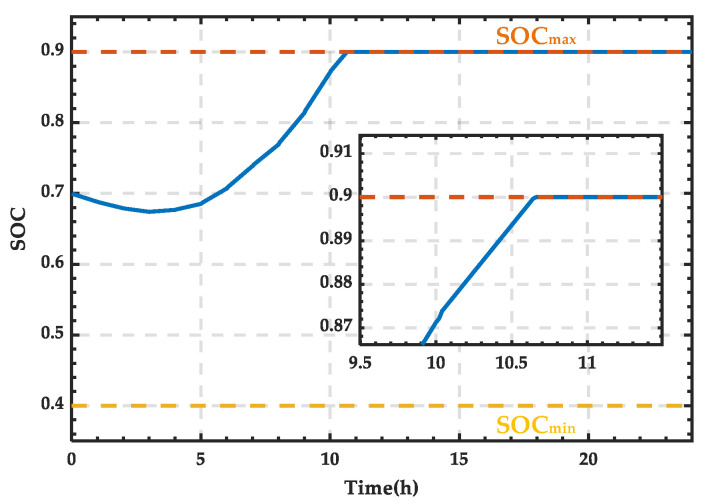
SOC variation curve of the ESS for a typical day in spring.

Based on the wind–PV-ESS coordinated configuration scheme, the SOC curve of the ESS can be obtained, as shown in Figure 8. Due to the set range limit on the SOC, it reaches a maximum value of 0.9 in this study, after which any excess energy is delivered to the external grid. As observed, the SOC curve briefly decreases at the beginning and then starts to rise, reaching the maximum value of 0.9 around 10:40, and thereafter it remains constant at this value.

**Figure 9:**
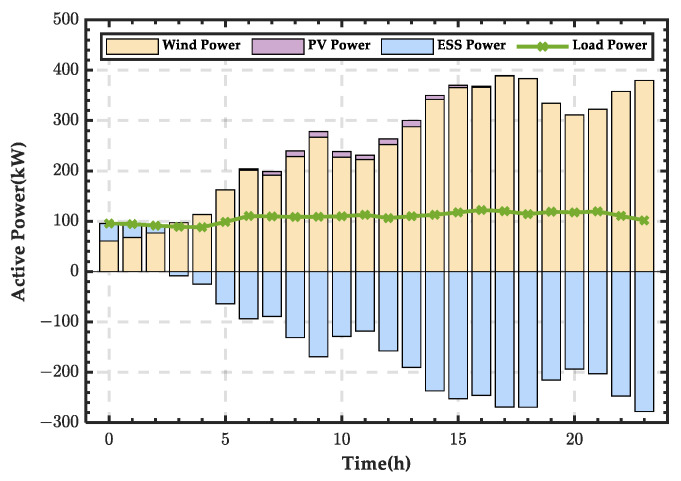
Power supply curve of the external grid for a typical day in spring.

As shown in Figure 8, after 10:40, the microgrid delivers the excess power to the external grid. The power supply curve of the external grid is shown in Figure 9, where negative values represent power being fed back into the grid. By performing integration, the amount of energy transferred is calculated to be 2923.39 kWh.

**Figure 10:**
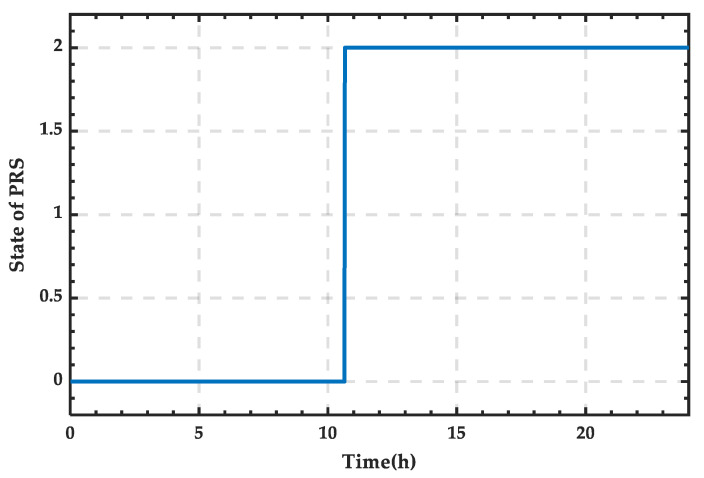
State change curve of PRS for a typical day in spring.

Based on Figure 9, the state change curve of the PRS can be further obtained, as shown in Figure 10. It can be observed that in the absence of power exchange, the PRS is in state 0. After 10:40, when the SCC operates in active inversion mode, the PRS is in state 2.

### 3.2. Typical Day in Summer

**Figure 11:**
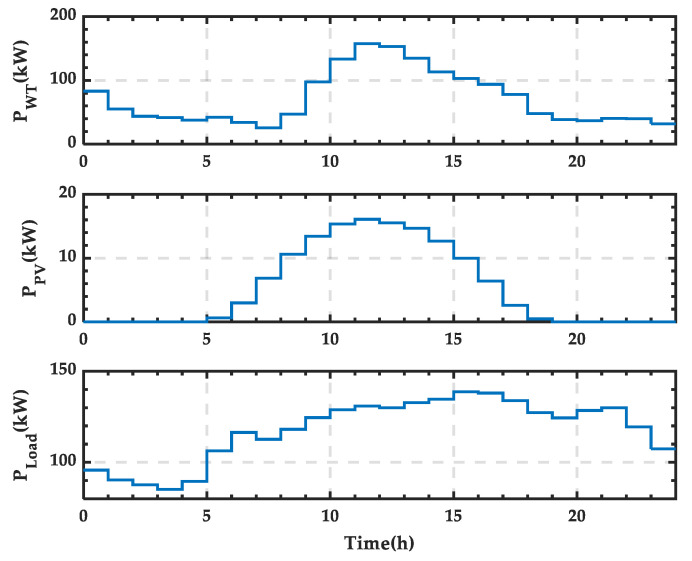
Wind and PV power output and load power curves for a typical day in summer.

This section selects the wind power output *P_WT_*, PV power output *P_PV_*, and load power consumption *P_Load_* data for a typical day in July as the study objects, dividing the day into 24 time periods. The power distribution for these 24 periods is shown in Figure 11. It can be observed that the wind and PV power outputs reach their maximum at noon, while the load power consumption is relatively low only during the early morning, with higher consumption during the rest of the day.

**Figure 12:**
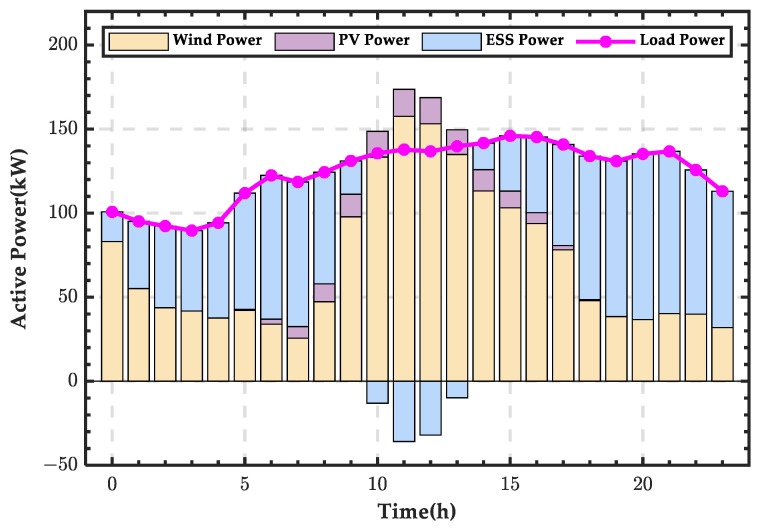
Wind–PV-ESS coordinated configuration scheme for a typical day in summer.

Based on the power balance equation of the microgrid, the wind–PV-ESS coordinated configuration scheme for a typical day in summer can be obtained, as shown in Figure 12. It is evident that the ESS predominantly needs to discharge to supplement the power deficit in the microgrid, except during the periods of high wind and PV output from 10:00 to 13:00, when it is in a charging state.

**Figure 13:**
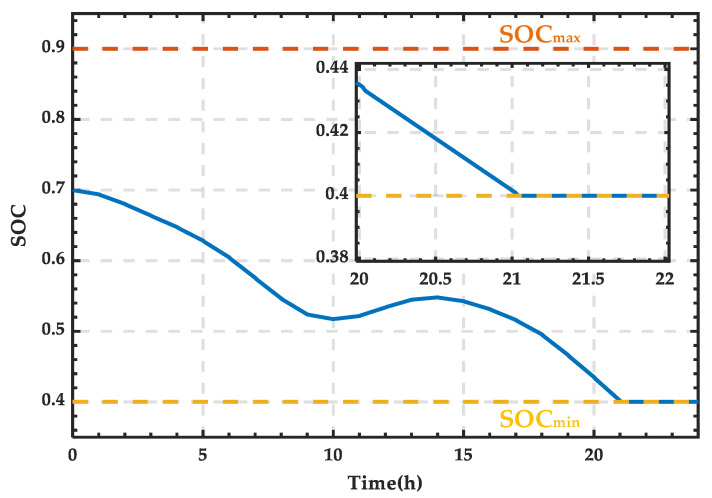
SOC variation curve of the ESS for a typical day in summer.

Based on the wind–PV-ESS coordinated configuration scheme, the SOC curve of the ESS is obtained, as shown in Figure 13. SOC variation curve of the ESS for a typical day in summer. It can be observed that the SOC increases only between 10:00 and 14:00. After 21:00, the SOC reaches its lower limit, requiring the SCC to operate in rectification mode to supply the necessary power for the microgrid.

**Figure 14:**
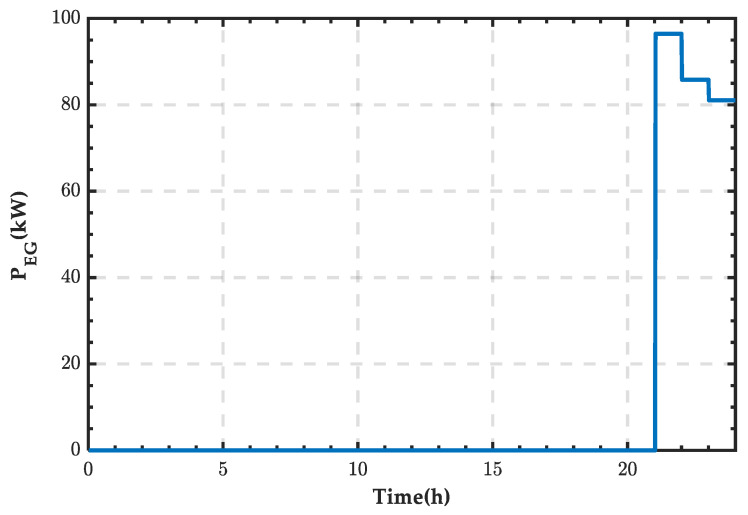
Power supply curve of the external grid for a typical day in summer.

From Figure 13, it can be observed that the microgrid requires an external energy supply between 21:00 and 24:00, while during other periods, the energy demand can be met by internal renewable energy and the storage system. The power supply curve of the external grid is shown in Figure 14, and integration calculations reveal that the supplied energy amounts to 261.38 kWh.

**Figure 15:**
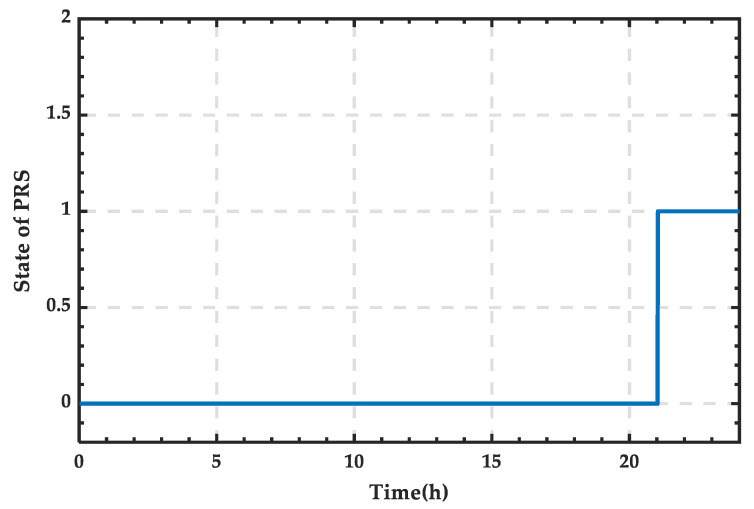
State change curve of PRS for a typical day in summer.

Based on Figure 14, the state change curve of PRS can be derived, as shown in Figure 15. It can be observed that the PRS remains in state 0 when there is no power exchange. When the SCC operates in rectification mode, the PRS changes to state 1.

### 3.3. Typical Day in Autumn

**Figure 16:**
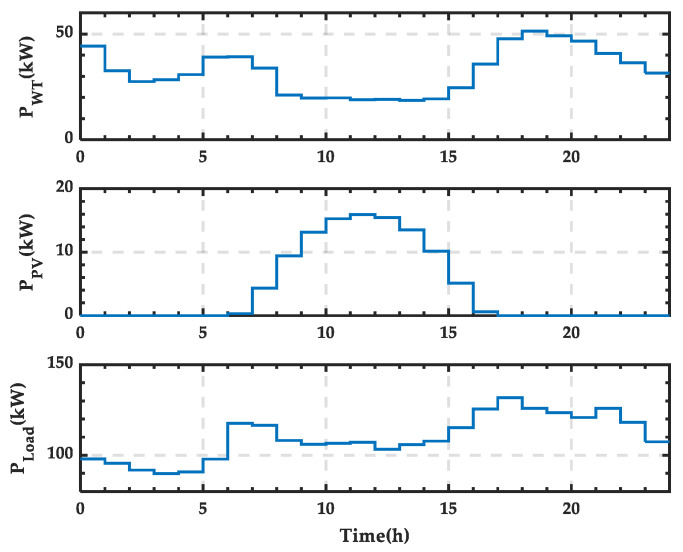
Wind and PV power output and load power curves for a typical day in autumn.

In this subsection, wind power output *P_WT_*, PV power output *P_PV_*, and load power consumption *P_Load_* data from a typical day in October are analyzed. The day is divided into 24 time periods, and the power distribution for each period is shown in Figure 16. It can be observed that wind power output is relatively low during the daytime, the PV power output reaches its peak at noon, and the load exhibits two consumption peak periods.

**Figure 17:**
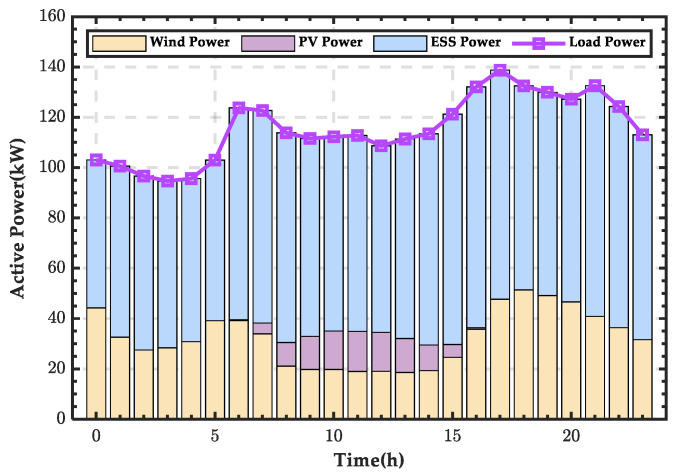
Wind–PV-ESS coordinated configuration scheme for a typical day in autumn.

Based on the microgrid power balance equation, the wind–PV-ESS coordinated configuration scheme for a typical autumn day is shown in Figure 17. It can be seen that on this typical day, the wind and PV power output are relatively low and cannot meet the power demand of load, necessitating the ESS to supplement the power deficit.

**Figure 18:**
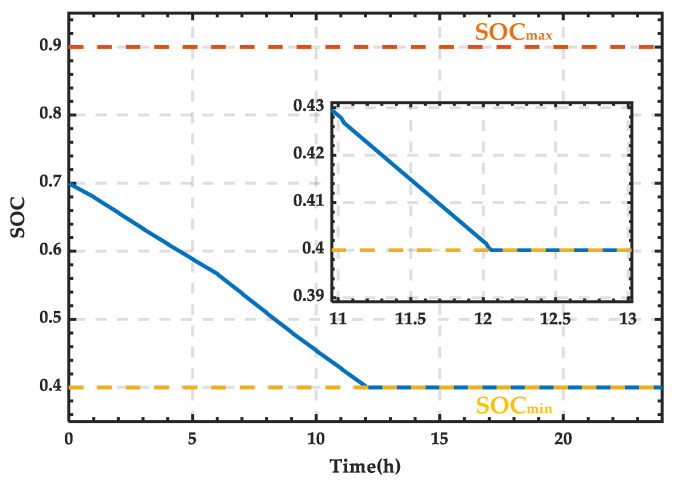
SOC variation curve of the ESS for a typical day in autumn.

Based on the wind–PV-ESS coordinated configuration scheme, the SOC curve of the ESS can be obtained, as shown in Figure 18. The SOC decreases to the lower limit of 0.4 around 12:00, after which external grid power is required to meet the power demand of the microgrid.

**Figure 19:**
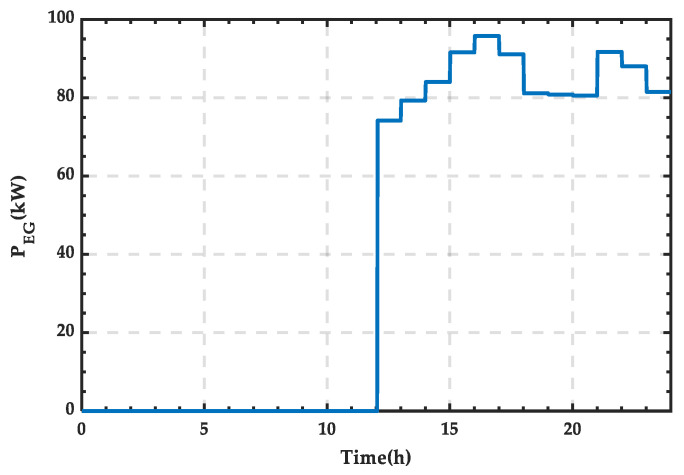
Power supply curve of the external grid for a typical day in autumn.

As shown in Figure 18, after 12:00, the external grid power is required, and the power supply curve is shown in Figure 19. Through integration, the total energy supplied by the external grid is calculated to be 1016.60 kWh.

**Figure 20:**
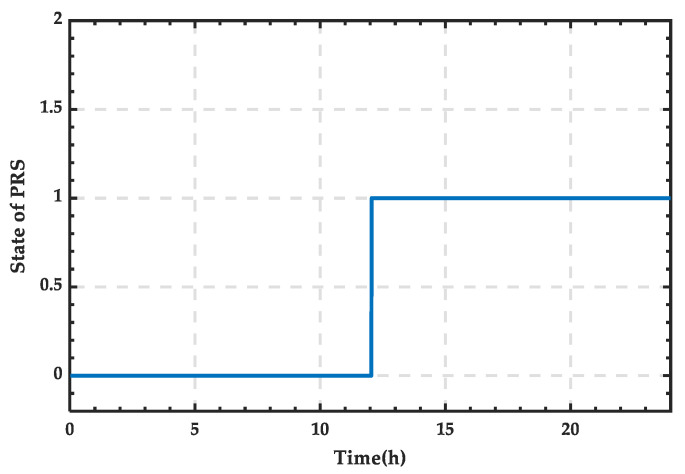
State change curve of PRS for a typical day in autumn.

Based on Figure 19, the state change curve of PRS can be further obtained as shown in Figure 20. It can be observed that in the absence of power exchange, the PRS is in state 0. After 12:00, when the SCC operates in rectification mode, the PRS is in state 1.

### 3.4. Typical Day in Winter

**Figure 21:**
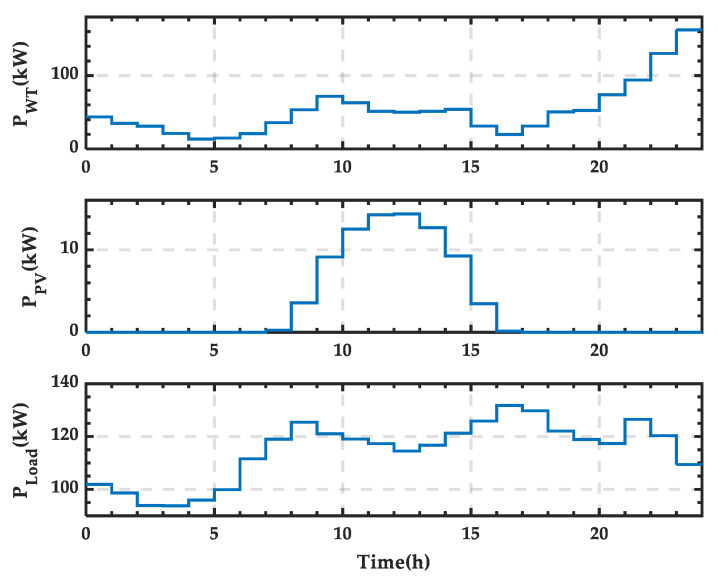
Wind and PV power output and load power curves for a typical day in winter.

This section selects the wind power output (*P_WT_*), PV power output (*P_PV_*), and load power consumption (*P_Load_*) data for a typical day in January. The day is divided into 24 time periods, and the power distribution for each period is shown in Figure 21. The wind power output peaks between 23:00 and 24:00 at night, while the PV power output peaks at noon. The load power consumption has multiple peak points, reaching a low consumption point in the early morning.

**Figure 22:**
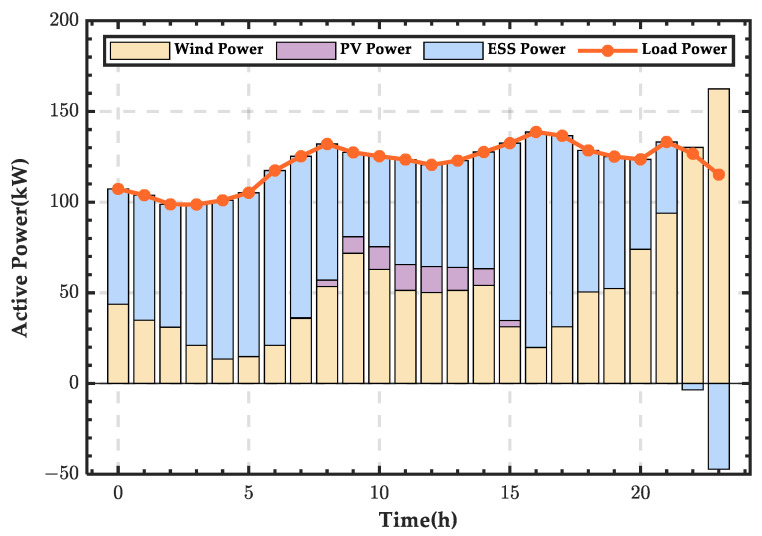
Wind–PV-ESS coordinated configuration scheme for a typical day in winter.

Based on the microgrid power balance equation, the wind–PV-ESS coordinated configuration scheme for the microgrid for a typical winter day is shown in Figure 22. It can be seen that during most periods, the ESS needs to discharge to supplement the energy deficit of the microgrid, and only during two night-time periods does it enter a charging state.

**Figure 23:**
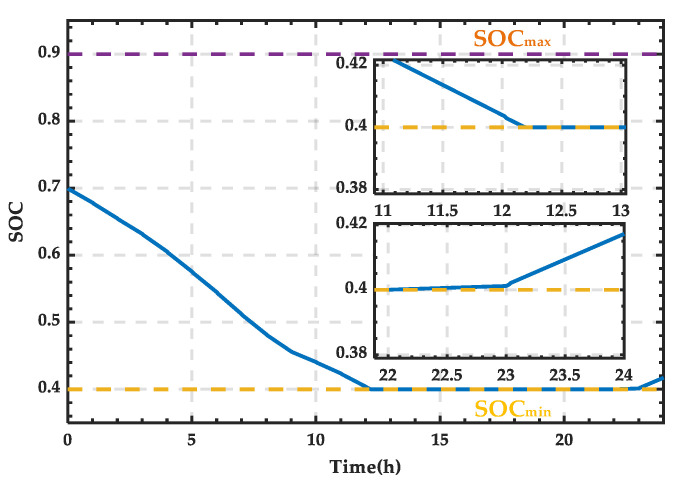
SOC variation curve of the ESS for a typical day in winter.

Based on the wind–PV-ESS coordinated configuration scheme, the SOC curve of the ESS can be obtained as shown in Figure 23. It can be observed that between 12:12 and 22:00, the external grid needs to supplement the energy. Furthermore, after 22:00, when the wind and PV output exceeds the demand, the trigger pulses of the SCC stop, and the ESS enters the charging state.

**Figure 24:**
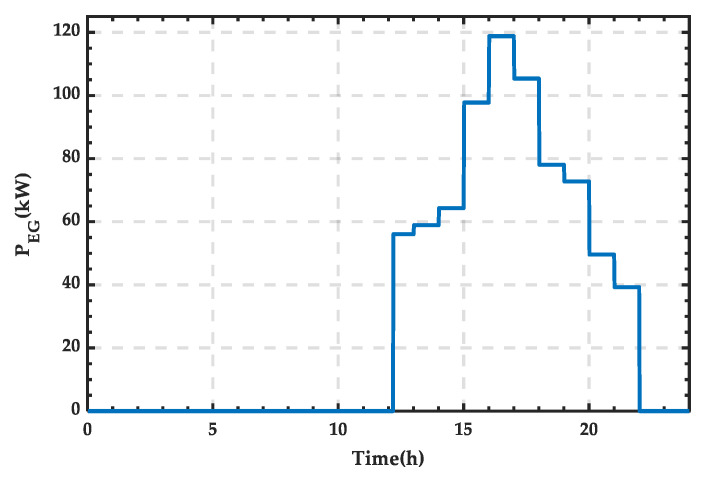
Power supply curve of the external grid for a typical day in winter.

As shown in Figure 23, external energy supply is required between 12:12 and 22:00. The power supply curve of the external grid is shown in Figure 24, and through integration, the corresponding amount of energy is 730.78 kWh.

**Figure 25:**
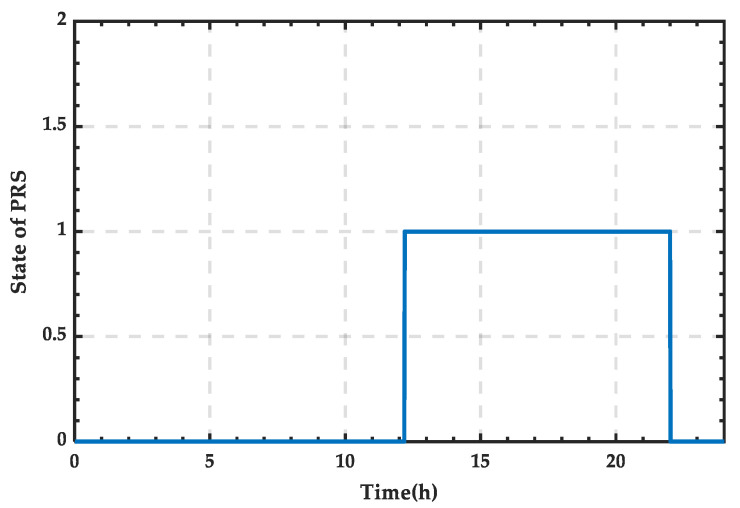
State change curve of PRS for a typical day in winter.

Based on Figure 24, the state variation curve of PRS can be obtained as shown in Figure 25. It can be observed that the PRS is in state 1 between 12:12 and 22:00, and in state 0 the rest of the time.

### 3.5. Comparison and Analysis of Microgrid Energy on Typical Days

Based on the data presented in Section 3.1, Section 3.2, Section 3.3, Section 3.4, the energy balance results of the microgrid on typical days in the four seasons are summarized in Table 2. This reflects the distribution of natural resources and load power consumption across different seasons. Among them, spring exhibits the richest wind resources, summer has the most abundant PV resources, and the load demand peaks in summer, while autumn requires the highest energy supply from the external grid.

## 4. Microgrid Simulation Analysis

**Figure 26:**
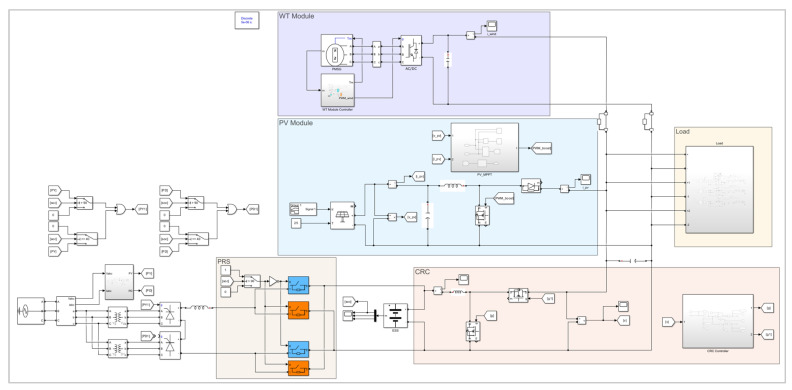
Image of the proposed microgrid structure implemented in Simulink.

As is evident from the above research, the states of the microgrid structure proposed in this paper can be classified into three types based on the states of the PRS. A simulation model is built on the Simulink platform with specific parameter settings for simulation analysis, validating the effectiveness of the proposed structure. The image of the microgrid structure proposed in this paper implemented in Simulink is shown as Figure 26, and each part corresponds with the topology shown in Figure 3, respectively.

In the simulation model presented in this paper, the PRS is set to position 1 when the microgrid is in states 0 and 1, and to position 2 when the microgrid is in state 2. As shown in Figure 26, the SOC serves as the detection signal. When the SOC is greater than 0.9 (“90” in Figure 26 represents 90%), the two orange switches are closed, and the two blue switches are open, simulating the state of the PRS being in position 2. Conversely, when the SOC is less than or equal to 0.9, the two orange switches are open, and the two blue switches are closed, thus simulating the switching process of the PRS from position 2 to position 1.

As shown Figure 26, a large number of power electronic devices are used in the Simulink simulation model. Table 3 provides a brief introduction to the names, parameters, and main function descriptions of each component.

### 4.1. Simulation Analysis When PRS Is in State 0

When the PRS is in state 0, indicating no energy exchange between the microgrid and the external grid, the load demand is fully met by energy storage, wind power, and PV power output. The simulation parameters of this microgrid in this state are shown in Table 3.

The scenario set in this subsection is as follows: From 0 to 1.5 s, wind and PV renewable energy sources simultaneously output electric energy. At 1.5 s, the wind power output drops to 0, while the PV power output remains unchanged. The specific simulation results are as follows.

**Figure 27:**
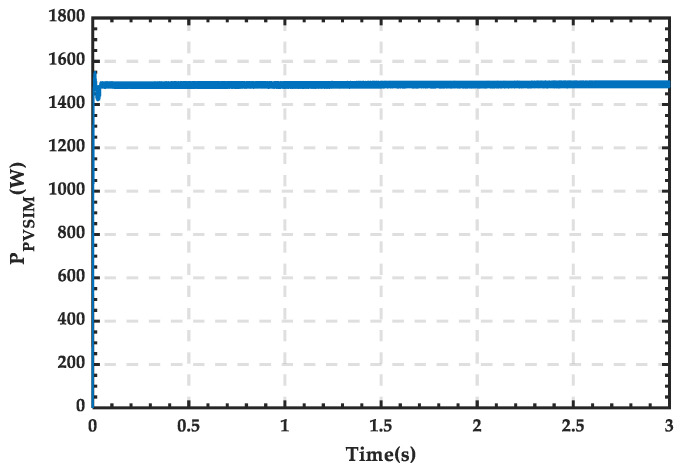
Power waveform generated by the PV module.

**Figure 28:**
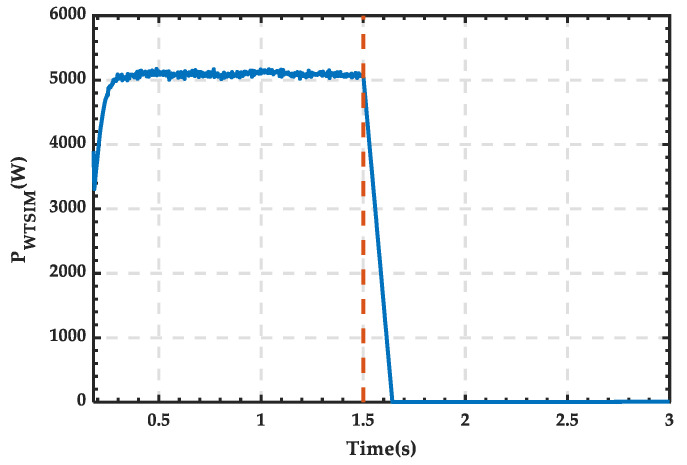
Power waveform generated by the WT module.

**Figure 29:**
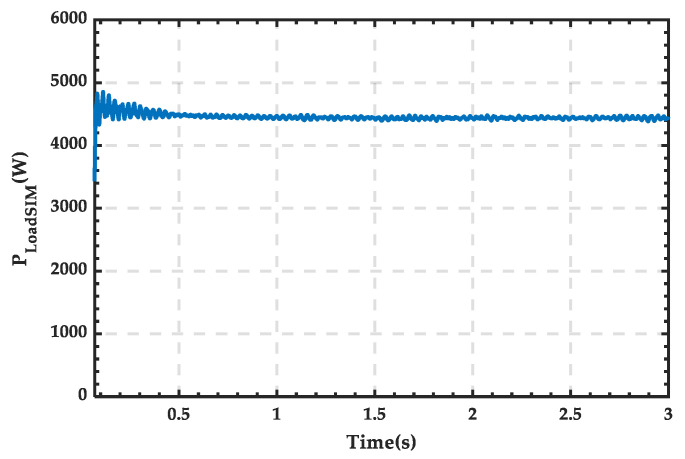
Power waveform of the load consumption.

The waveform of PV power output is shown in Figure 27, with a stable power value of 1500 W. The waveform of wind power output is shown in Figure 28, where the power output of the wind turbine remains stable at around 5100 W during normal operation, gradually decreasing to 0 after being shut down at 1.5 s. The waveform of load power consumption is shown in Figure 29, with the power stabilizing at around 4400 W.

**Figure 30:**
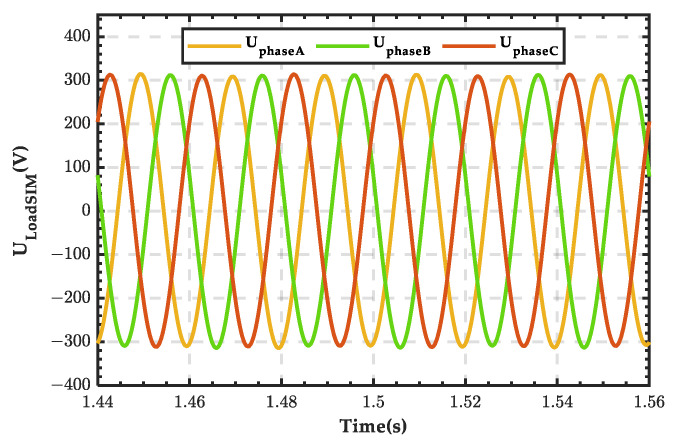
Phase voltage waveform of the microgrid load.

**Figure 31:**
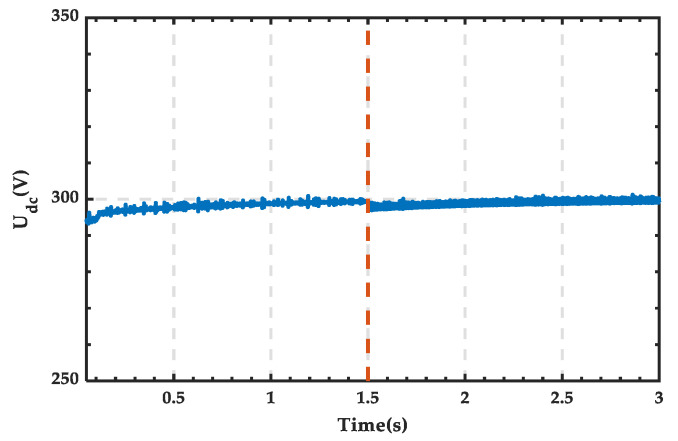
Voltage waveform of the DC bus. (The dashed line indicates the wind power output disconnected at 0.5 s).

The phase voltage waveform of the three-phase load is shown in Figure 30, with a voltage amplitude of 311 V, consistent with the set value, and no significant fluctuations occur before or after the change in wind power output. The waveform of the DC bus voltage is shown in Figure 31, with a stable voltage maintained at 300 V. After the wind power output is disconnected, there is a slight fluctuation, but it quickly returns to stability. The above analysis demonstrates that the microgrid structure proposed in this paper exhibits good stability.

**Figure 32:**
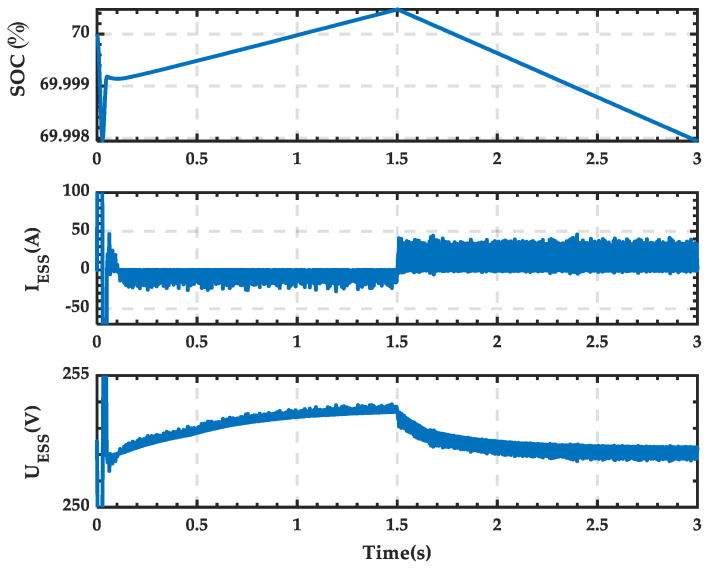
SOC, current, and terminal voltage waveforms of the ESS when PRS is in state 0.

The waveforms of the SOC, current, and terminal voltage of the ESS in the microgrid are shown in Figure 32. It can be seen that from 0 to 1.5 s, the SOC increases, the current is negative, and the energy storage is in the charging state, with a slight increase in terminal voltage. From 1.5 s to 3.0 s, the SOC decreases, the current is positive, and the energy storage is in the discharging state, with a slight decrease in terminal voltage.

### 4.2. Simulation Analysis When PRS Is in State 1

When the SOC of the ESS is detected to be lower than the lower limit SOC_min_, the state of the PRS changes to state 1 and the switch contact is in position 1. In this state, the SCC operates in rectification mode, and the external grid will supply power through the SCC. The simulation parameters for the microgrid in this state are shown in Table 3. The trigger angle of the SCC is set to 30° and the initial SOC of the ESS is set to 39.97%. In addition, to facilitate the observation of energy interaction between the ESS and the external grid through the PRS and SCC, no trigger pulse is provided to the CRC circuit.

**Figure 33:**
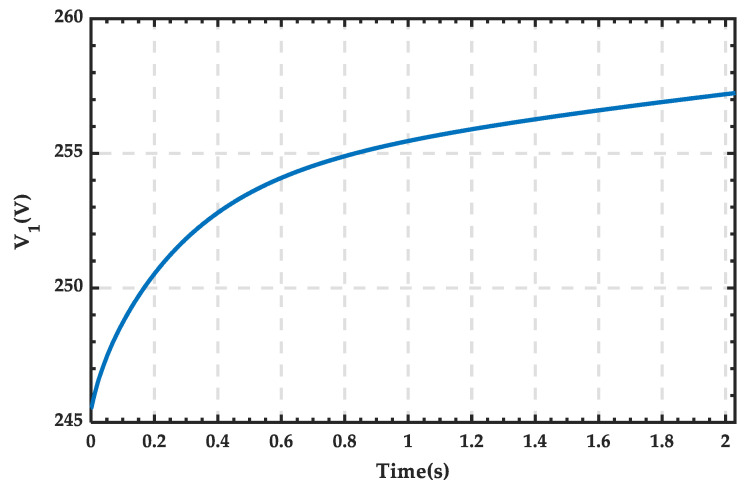
Rectifier outlet voltage waveform.

**Figure 34:**
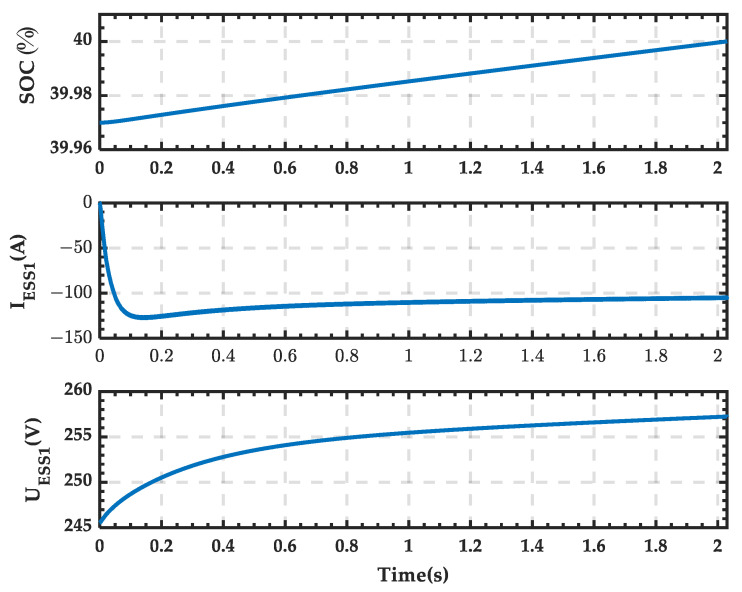
SOC, current, and terminal voltage waveforms of the ESS when PRS is in state 1.

The rectifier outlet voltage V_1_ is shown in Figure 33, where the voltage value is positive, and the polarity of the voltage is consistent with the set positive direction. The waveforms of the SOC, current, and terminal voltage of the ESS in the microgrid are shown in Figure 34. The current is negative, and the voltage gradually increases. After 2.04 s, the SOC increases from 39.97% to 40%.

When the SOC of the ESS reaches the preset lower limit of 0.4, the state of the PRS changes to state 0. According to the simulation settings in this paper, the contact of the PRS remains at position 1. However, the trigger pulse to the SCC will cease, and the SCC will no longer supply electric energy to the DC side. The simulation results are as follows.

**Figure 35:**
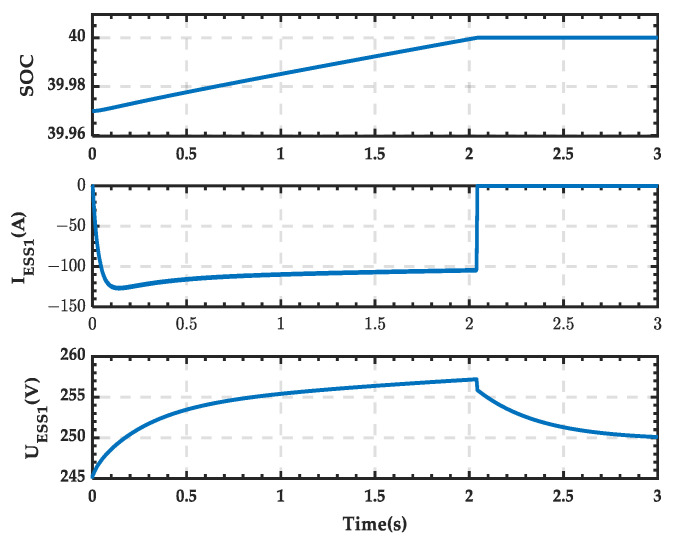
SOC, current, and voltage waveforms of the ESS when PRS switches from state 1 to state 0.

The waveforms of the SOC, current, and terminal voltage of the ESS in the microgrid during the transition process are shown in Figure 35. It can be observed that the SOC remains unchanged after reaching the lower limit of 0.4, the current drops to 0, and the voltage gradually decreases to the nominal voltage.

**Figure 36:**
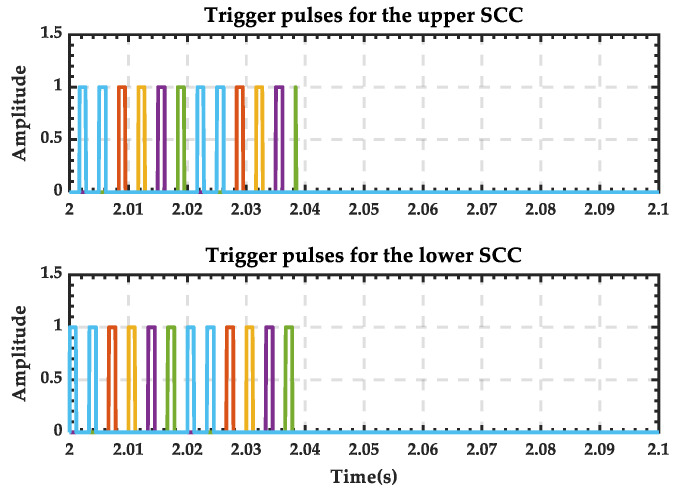
Trigger pulses of the twelve-pulse SCC when PRS switches from state 1 to state 0. (The six colors correspond to the six triggering pulses of SCC).

**Figure 37:**
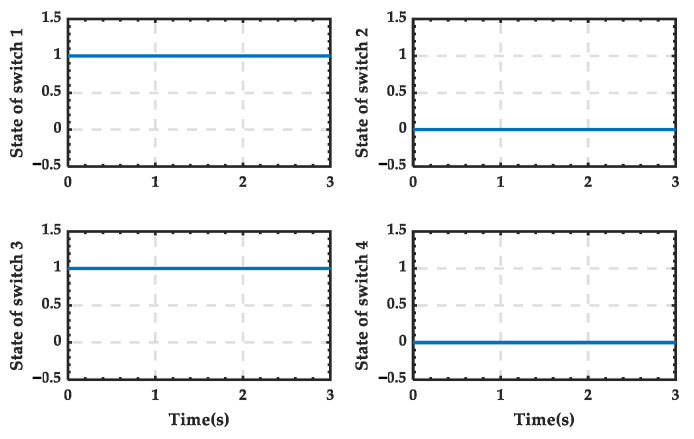
State of the switches when PRS switches from state 1 to state 0.

The trigger pulse waveform of the 12-pulse SCC during the transition process is shown in Figure 36, and it can be seen that there is no pulse output after 2.04 s. And the trigger pulses supplied to the switches simulating the PRS are shown in Figure 37. It is evident that switches 1 and 3 (depicted as blue in Figure 26) remain closed throughout, while switches 2 and 4 (depicted as orange in Figure 26) remain open. This indicates that the contact of the PRS consistently remains at position 1, which aligns with the analysis.

### 4.3. Simulation Analysis When PRS Is in State 2

When the SOC of the ESS exceeds the upper limit SOC_max_, the state of PRS changes to state 2. The SCC operates in active inversion mode, and excess wind and PV power from the microgrid is sent to the external grid through the SCC. The simulation parameters for the microgrid in this state are shown in Table 3. The trigger angle of the SCC is set to 120° and the initial SOC of the ESS is set to 90.04%.

**Figure 38:**
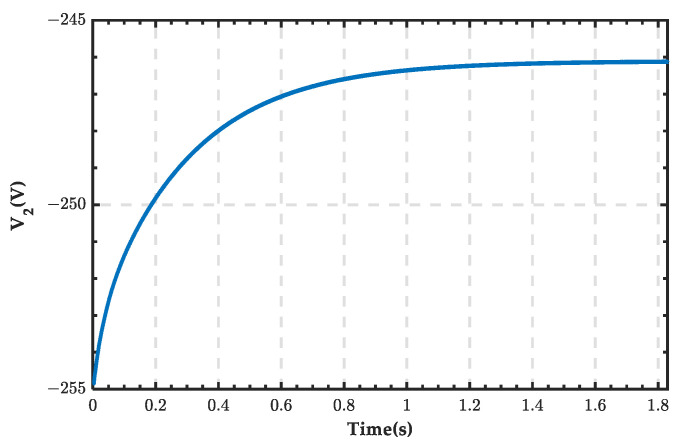
Inverter outlet voltage waveform.

**Figure 39:**
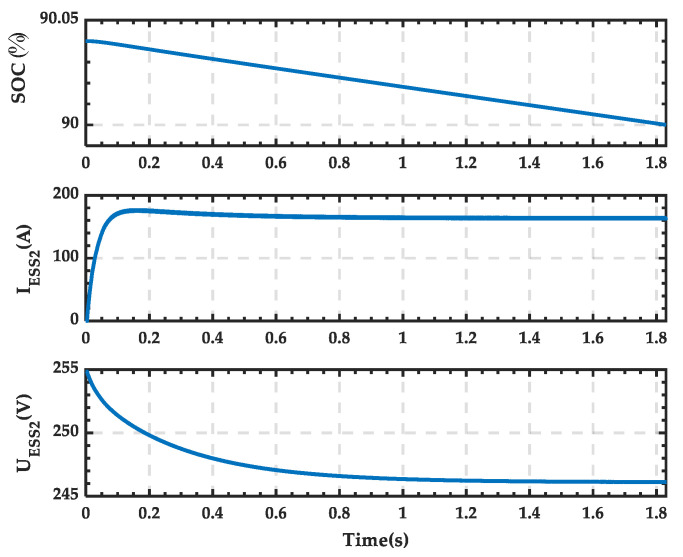
SOC, current, and terminal voltage waveforms of the ESS when PRS is in state 2.

The inverter outlet voltage waveform is shown in Figure 38. The voltage is negative, indicating that the voltage polarity is opposite to the set positive direction. The waveforms of the SOC, current, and terminal voltage of the ESS in the microgrid are shown in Figure 39. The current is positive, and the voltage gradually decreases. After 1.84 s, the SOC decreases from 90.04% to 90%.

When the SOC of the ESS reaches the preset upper limit of 0.9, the state of the PRS changes from state 2 to state 0. According to the simulation settings in this paper, the contact of the PRS will shift from position 2 to position 1. Simultaneously, the trigger pulse for the SCC will cease, causing the SCC to stop sending excess electric energy from the microgrid to the external grid. The simulation results for this process are as follows.

**Figure 40:**
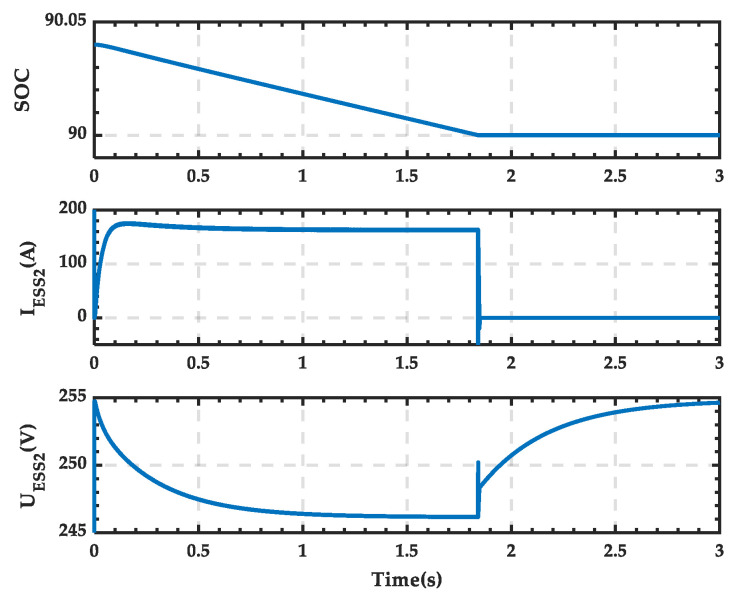
SOC, current, and voltage waveforms of the ESS when PRS switches from state 2 to state 0.

The waveforms of the SOC, current, and terminal voltage of the ESS in the microgrid during the transition process are shown in Figure 40. It can be observed that the SOC remains unchanged after decreasing to the upper limit of 0.9, the current drops to 0, and the voltage gradually increases.

**Figure 41:**
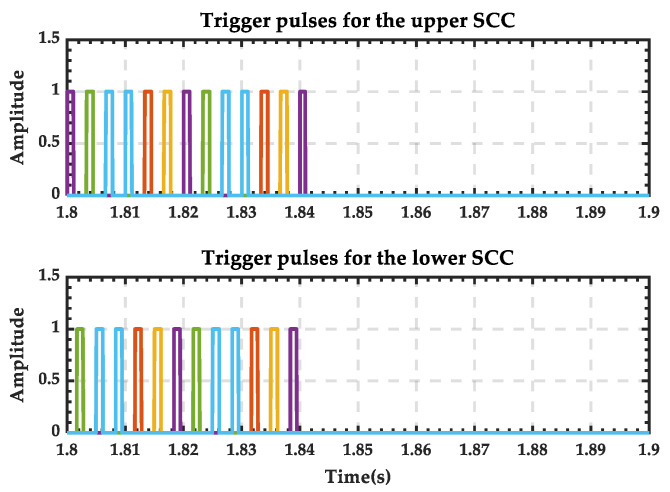
Trigger pulses of the twelve-pulse SCC when PRS switches from state 2 to state 0.

**Figure 42:**
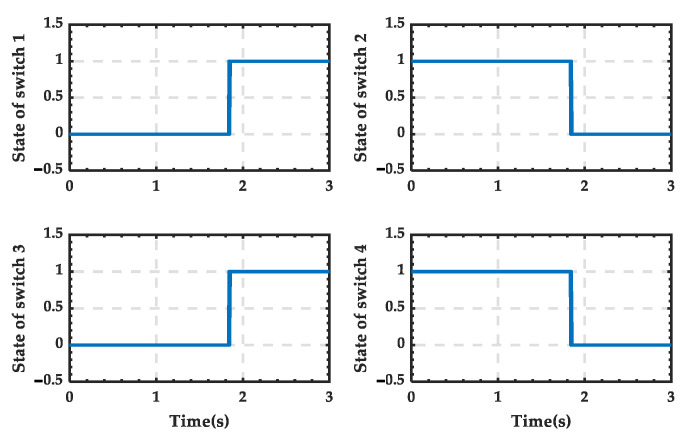
State of the switches when PRS switches from state 2 to state 0.

The trigger pulse waveform of the 12-pulse SCC during the transition process is shown in Figure 41, and it can be seen that there is no pulse output after 1.84 s. And the trigger pulses supplied to the switches simulating the PRS are shown in Figure 42. It is evident that switches 1 and 3 (depicted as blue in Figure 26) change from open to closed, while switches 2 and 4 (depicted as orange in Figure 26) change from closed to open. This indicates that the contact of the PRS shifts from position 2 to position 1, which is consistent with the analysis.

## 5. Comparisons Between the Proposal Structure and Traditional Structures

**Figure 43:**
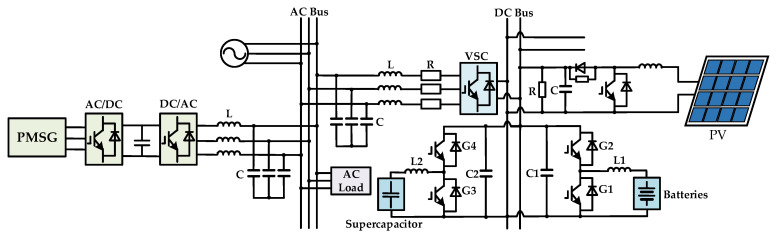
Topology of the conventional AC/DC hybrid microgrid.

In this section, this paper will initially present comparisons between the newly proposed structure and traditional structures, as detailed below:

(1) Reduction in Economic Costs: The proposed AC/DC hybrid microgrid structure based on the SCC and PRS effectively utilizes the capabilities of SCC for both rectification and active inversion. Compared to the conventional microgrid structure as shown in Figure 43 [26], the distributed wind module is directly connected to the DC grid, minimizing the DC/AC conversion process, which not only reduces costs, but also decreases energy losses. The efficient use of renewable energy sources such as wind and PV energy reduces electricity purchase costs from the external grid. Additionally, in comparing the equipment costs of VSC and SCC, we primarily focus on the following aspects based on practical engineering experience:

Procurement Costs: VSC involves fully controlled power electronic devices such as IGBT, which require advanced technology and are relatively expensive. They also necessitate complex control and protection systems. A medium-sized VSC device for microgrids has a procurement cost in the range of hundreds of thousands of RMB. In contrast, SCC employs semi-controlled devices with a simple structure and control, resulting in a procurement cost of tens of thousands of RMB.

Installation and Commissioning Costs: VSC demands sufficient installation environments, necessitating specialized grounding, cooling, and anti-interference measures. Installation is challenging, and commissioning is complex. Conversely, SCC installation is relatively straightforward, with lower environmental requirements and easier commissioning, leading to lower installation and commissioning costs. Generally, the installation and commissioning costs of VSC may be 20–50% higher than those of SCC.

Operation and Maintenance Costs: The fully controlled devices in VSC operate at high frequencies, resulting in significant switching losses and high demands for cooling and complex control systems. The probability of failures is relatively high, requiring professional maintenance with high technical requirements and costs. In contrast, the semi-controlled devices in SCC operate at lower frequencies with minimal losses, providing high operational stability and simpler, lower-cost maintenance. Estimates suggest that the annual operation and maintenance cost of VSC may be 1.5–3.5% of the equipment procurement cost, compared to 1.0–2.5% for SCC.

(2) Improvement of Converter Efficiency: Existing microgrid structures typically use VSC to connect the AC side and DC side. The losses incurred by VSC operating at high frequencies are non-negligible. Data from [27] indicate that the turn-on energy loss per pulse and turn-off energy loss per pulse of the IGBT are 19 mJ and 12 mJ, respectively. At a switching frequency of 5 kHz, the switching loss amounts to 165 W. In contrast, the switching loss of SCC is virtually negligible. The difference between the two is substantial, especially in microgrids. Although the harmonic content of a 12-pulse SCC is still higher than that of a VSC, the THD can be suppressed to below 3% by installing simple passive filters. However, the high-frequency switching of VSC tends to generate broadband harmonics, necessitating the installation of large-capacity filters or active damping. The losses associated with these filters are significant, and simultaneously, high-frequency harmonics can also increase eddy current losses in cables and transformers. Therefore, the structure proposed in this paper exhibits higher efficiency compared to conventional structures.

(3) Enhanced Structural Stability: In traditional microgrid structures, wind turbines are typically connected to the AC side via a two-stage AC-DC-AC converter. This proposed structure connects wind turbines directly to the DC bus, eliminating the inversion stage, which improves power quality. The DC bus relies on the control of CRC to maintain voltage stability and make the microgrid less susceptible to the fluctuations of the external grid compared to traditional AC microgrids.

(4) Improved Utilization of Renewable Energy: Due to the mismatch between renewable energy generation and load demand, the existing microgrids often face significant wind and PV power curtailment. The proposed structure can fully utilize wind and PV power, enhancing the consumption capacity of renewable energy, benefiting environmental protection.

**Figure 44:**
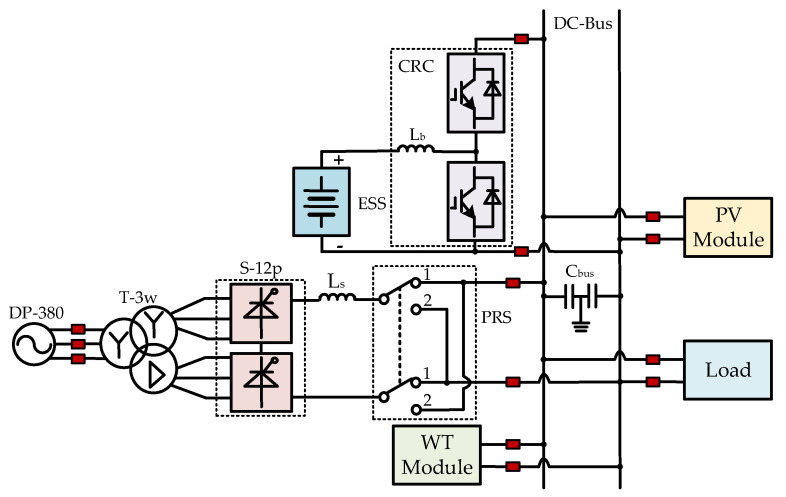
Topology of the structure using interlinking converters.

Next, this paper will conduct a comparative analysis between the proposed structure and the structure presented in Figure 44. The structure shown in Figure 44 can be regarded as a variation in the proposed structure, with the difference being that the ESS and CRC components, which are originally located between the PRS and the DC bus, have been removed, and instead connected directly to the DC bus as a single module.

(1) Economy: The structure proposed in this paper can be more compactly integrated, reducing the need for additional connecting components, such as the elimination of the requirement for complex interface equipment specifically configured for the DC bus, which also lowers maintenance costs. The structure shown in Figure 44 requires high-quality insulating materials and protective devices for the DC bus, and may additionally necessitate isolation equipment, thereby increasing investment and maintenance costs. Furthermore, the structure proposed in this paper can flexibly regulate the energy flow between the AC and DC sections through CRC circuit, whereas in the structure shown in Figure 44, energy losses occur during the conversion process between the AC and DC sections due to the necessity of passing through the DC bus, increasing operational costs.

(2) Service Quality: In the structure proposed in this paper, the CRC circuit can precisely control the voltage of the DC bus, and the ESS can absorb harmonic currents on the AC side, improving power quality. In contrast, harmonics on the AC side in the structure shown in Figure 44 are directly injected into the DC bus, affecting power quality. The proposed structure can also quickly adjust voltage and current, exhibiting a faster response time, whereas the structure shown in Figure 44 has a delay.

(3) Reliability: The ESS and CRC can serve as isolation modules, allowing for rapid disconnection of the fault source when a fault occurs in either the DC or AC section, preventing the fault from spreading throughout the microgrid. The structure shown in Figure 44 lacks this functionality.

Overall, the above analysis demonstrates that the proposed AC/DC hybrid microgrid structure offers substantial advantages in terms of economy, efficiency, service quality, and reliability compared to traditional or existing microgrid structures.

## 6. Conclusions

This paper designs a novel AC/DC hybrid microgrid structure based on the SCC and PRS, taking advantage of the ability of the SCC to perform both rectification and active inversion. The control strategy of this structure when PRS is in different states is also introduced. Based on the microgrid structure, this paper studies the coordinated configuration schemes for wind, PV, and ESS power on typical days of the four seasons (spring, summer, autumn, and winter). It also analyzes the state changes in the PRS during these typical days and compares the energy balance results across different seasons, analyzing the distribution of resources. Additionally, this paper sets specific parameters and conducts simulation analysis on the microgrid under different states of the PRS, verifying the feasibility of the microgrid. Finally, this paper conducts a comparative analysis between the proposed structure and traditional microgrid structures.

Moreover, the structure proposed in this paper also exerts an influence on the frequency, inertia, and multi-energy interactions of the power system. This paper only provides the following analysis, and in-depth discussions require further research to be obtained:

(1) In terms of frequency, SCC can quickly adjust the power, which is beneficial for maintaining the frequency stability of the AC section. The coordination between the PRS and ESS enables rapid charging and discharging during frequency fluctuations, enhancing the frequency regulation ability.
(2) In terms of inertia, the combined use of the CRC and ESS can achieve rapid adjustment of the DC bus voltage, thereby simulating a virtual inertia effect. When there are power fluctuations, the ESS can respond quickly and provide the necessary power support, thus improving the dynamic response performance of the system.
(3) In terms of multi-energy interactions, the introduction of SCC and PRS makes the energy flow between the AC and DC sections more flexible and controllable. The microgrid can flexibly integrate different types of distributed energy sources and ESSs. By adjusting the control strategy, the optimal scheduling and utilization of these energy sources can be achieved, thus enhancing the overall flexibility and reliability of the system.

Certainly, the research conducted in this paper also has the following limitations: Only wind and PV energy are considered in this study, but other energy sources such as biomass energy can be investigated in subsequent research. During the transition of the PRS states, there may be significant transient currents in practical application scenarios, which need to be further considered when constructing the microgrid for specific applications. Additionally, the research in this paper has high reliability requirements for PRSs, and it is necessary to research and develop high-performance switches to enhance the robustness of the system.

## Figures and Tables

**Table 1:** Operating states and conditions considered for the microgrid.

Operational State	Conditions
State 0	SOC ∈ [SOC_min_, SOC_max_], no trigger pulse applied to the SCC
State 1	SOC < SOC_min_, α < 90°
State 2	SOC > SOC_max_, α > 90°

**Table 2:** Energy balance results of the microgrid on typical days.

Typical Day	Wind Power Energy (kWh)	PV Energy (kWh)	ESS Energy (kWh)	External Grid Energy (kWh)	Load Energy (kWh)
Spring	6006.13	91.67	−586.15	−2923.39	2458.85
Summer	1711.13	128.35	879.28	261.38	2831.13
Autumn	776.04	103.25	879.82	1016.60	2636.93
Winter	1256.14	79.62	830.03	730.78	2751.75

**Table 3:** Description of each component used in the Simulink model.

Number	Component Name	Parameters		Main Function Description
1	Three-Phase Source	Phase-to-phase voltage Phase angle of phase A	380 V 0 °	To simulate the external power grid supply.
2	Three-Phase VI Measurement	Voltage measurement Current measurement	phase-to-ground yes	To measure the three-phase voltage and current signals output by the power supply.
3	Three-Phase Transformer (Two Windings)	Nominal power Frequency V_1_/V_2_ Connection	1 × 10^4^ VA 50 Hz 380/130 V Yg/Yg (Yg/D1)	To perform voltage transformation and power transmission.
4	Universal Bridge (SCR)	Number of bridge arms Forward voltage V_f_	3 1.2 V	To perform the conversion between AC and DC power, and to enable energy interaction between the DC and AC sections of the microgrid.
5	Pulse Generator (SCR)	Generator type Winding connection	12-pulse D1	To generate the triggering pulses required for the 12-pulse SCC.
6	Inductance L_s_	Inductance	10 mH	To filter and stabilize the output DC voltage.
7	Ideal Switch	Internal resistance R_on_ Initial state	1 mΩ 0	To simulate the function of the PRS.
8	Battery	Type Nominal voltage Rated capacity Batterty response time	Lead–Acid 250 V 200 Ah 1 s	To simulate the Lead–Acid battery.
9	Mosfet	FET resistance R_on_ Diode resistance R_d_	0.1 Ω 0.01 Ω	To achieve on/off control of the current under the control of the input signal.
10	Inductance L_b_	Inductance	3 mH	To store energy and achieve the function of voltage boosting.
11	CRC Controller	K_P_ K_i_ Saturation1 Saturation2 Repeating sequence	6 8 [−0.2, 11] [−11, 0.2] [0, 5 × 10^−4^]	To enable bidirectional energy interaction between the ESS system and the DC section.
12	Capacitance C_bus_	Capacitance	16,000 μF	To store energy and smooth fluctuations in the DC-bus voltage.
13	PV Array	Terminal voltage Temperature	250 V 25 °C	To convert PV energy into electric energy.
14	Capacitance C_pv_	Capacitance	100 μF	To filter and stabilize the DC voltage output from PV array.
15	Inductance L_pv_	Inductance	0.2 H	To store power and achieve the function of voltage boosting.
16	Diode	Resistance R_on_ Forward voltage V_f_	1 mΩ 0.8 V	To isolate, continue current flow, and prevent backflow current.
17	PWM Generator (DC-DC)	Switching frequency	5000 Hz	To provide trigger pulses for the MOSFET of the BOOST circuit.
18	Load	V_load_ R_loadA_, R_loadC_ R_loadB_	220 V 30 Ω 40 Ω	To serve as the loads connected to the DC bus of the microgrid.
19	Breaker	Breaker resistance R_on_ Snubber resistance R_s_ Snubber capacitance	0.01 Ω 1 × 10^6^ Ω inf	To disconnect the wind module from the DC bus in order to simulate the loss of wind energy.
20	PMSG	Stator phase resistance Armature inductance Voltage constant Wind speed	5 mΩ 2 mH 1451 7 m/s	To convert mechanical energy into electric energy.
21	Universal Bridge (IGBT)	Number of bridge arms Forward voltage [V_f_ V_fd_]	3 [1.2 V 0.8 V]	To rectify and achieve the transmission of wind turbine electric energy to the DC bus.
22	Current Measurement	—	—	To measure the current signals.
23	Voltage Measurement	—	—	To measure the voltage signals.
24	Scope	—	—	To display and record signals during the simulation process.

## Data Availability

The data presented in this study are available on request from the corresponding author.
